# *In vitro* and *in vivo* activities of DW-3-15, a commercial praziquantel derivative, against *Schistosoma japonicum*

**DOI:** 10.1186/s13071-019-3442-7

**Published:** 2019-05-03

**Authors:** Xiaoli Wang, Dan Yu, Chunxiang Li, Tingzheng Zhan, Tingting Zhang, Huihui Ma, Jing Xu, Chaoming Xia

**Affiliations:** 1 Department of Parasitology, Medical College of Soochow University, 199 Renai Road, Suzhou, 215123 China; 2 Department of Microbiology and Parasitology, Anhui Key Laboratory of Infection and Immunity, Bengbu Medical College, 2600 Donghai Road, Bengbu, 233030 China; 3 Department of Parasitology, Guangxi Medical University, 22 Shuangyong Road, Nanning, 530021 China

**Keywords:** Praziquantel, *Schistosoma japonicum*, Schistosomicide

## Abstract

**Background:**

Schistosomiasis is a debilitating neglected tropical disease that affects approximately 190 million people around the world. Praziquantel (PZQ) is the only drug available for use against all *Schistosoma* species. Although PZQ has a high efficacy, recognized concerns have prompted the development of new, alternative drugs for repeated use in endemic areas where PZQ efficacy against strains of *Schistosoma* is reduced. A hybrid drug containing different pharmacophores within a single molecule is a promising strategy. Our earlier *in vivo* studies showed the significant antiparasitic activity of a praziquantel derivative, DW-3-15, against *Schistosoma japonicum*. In the present study, DW-3-15 was synthesized in large amounts by a pharmaceutical company and its schistosomicidal efficacy and stability were further confirmed. Parameters such as parasite viability, pairing and oviposition were evaluated *in vitro*. An *in vivo* study was conducted to assess the effect of commercial DW-3-15 on worm burden, egg production and diameter of granulomas. Additionally, to gain insight into the mechanism of action for DW-3-15, morphological changes in the tegument of *S. japonicum* were also examined.

**Results:**

The *in vitro* study showed the antiparasitic activity of DW-3-15 against *S. japonicum*, with significant reductions in viability of adult and juvenile worms, worm pairings and egg output. Compared to PZQ, DW-3-15 induced similar ultrastructural changes and evident destruction of the tegument surface in male worms. *In vivo*, the oral administration of DW-3-15 at a dose of 400 mg/kg per day for five consecutive days in mice significantly reduced the total worm burden and number of eggs in the liver. Histological analysis of the livers showed a marked reduction in the average diameter of the egg granuloma.

**Conclusions:**

Our findings suggest that DW-3-15, a PZQ derivative with the prospect of commercial production, can be developed as a potential promising schistosomicide.

**Electronic supplementary material:**

The online version of this article (10.1186/s13071-019-3442-7) contains supplementary material, which is available to authorized users.

## Background

Schistosomiasis is one of the most harmful parasitic diseases in many tropical and sub-tropical regions. The World Health Organization (WHO) estimates that approximately 190 million people are infected with the disease and a further 700 million are at a high risk of infection [1–3]. In 2016, the global morbidity burden of schistosomiasis was calculated as approximately 1.86 million disability-adjusted life years (DALYs), but the actual burden is thought to be underestimated [4–6]. *Schistosoma haematobium*, *S. japonicum* and *S. mansoni* are the main *Schistosoma* species parasitizing humans. The disease is caused primarily by the deposition of parasite eggs in the intestines, liver, bladder and other major organs, and followed by the formation of granulomas. In advanced cases, *Schistosoma* infections can result in severe manifestations characterized by hepatic fibrosis and, depending on the species, either intestinal fibrosis or genitourinary tract calcification and ureteric and bladder fibrosis [7].

For the past 40 years, praziquantel (PZQ), which can treat all species of schistosomiasis, has been the only recommended drug for this devastating disease. Although PZQ is particularly effective against *Schistosoma* worms at the adult stage, it has limited efficacy against schistosomula and juvenile adults [8]. Long-term, repeated, mass deployment of PZQ as ‘preventive chemotherapy’ in endemic areas may serve to accelerate drug resistance or tolerance. *S. mansoni* and *S. japonicum* strains showing resistance or insensitivity to PZQ have been reported in laboratories and sporadic reports of reduced susceptibility have come from the field [9–12]. Consequently, novel antischistosomal drugs to replace PZQ should be procured in advance of PZQ inefficacy. Important advances have been made in either repurposing existing drugs originally used to treat other diseases, or developing new compound series [13–15], and large-scale screening for drug repurposing strategy has led to the discovery of new antischistosomal candidates [16]. Along with molecular target-specific agents, some inhibitors targeting schistosomal enzymes have been reported to exhibit significant schistosomicidal effects [17, 18]. Additionally, other natural, chemotherapeutic compounds with antischistosomal activities and uncertain mechanisms of action have been identified [19, 20]. However, considering the high failure rate of these candidate compounds during pre-clinical and clinical trials, the development of alternative drugs requires further significant investment of time and resources.

Although malaria and schistosomiasis affect different tissues, they share a common hematophagous feature, i.e. free heme constitutes a major threat for both parasites [21]. Therefore, some anti-malarial drugs reported to interfere with heme metabolism have been tested for potential schistosomicidal activity. Among these, artemisinin analogs have been shown to possess significant efficacy against *S. haematobium* and *S. mansoni*, targeting the schistosomula in particular [22, 23] and moderately reducing adult worm burdens [24, 25]. Similarly, trioxaquine PA1259, a hybrid drug based on a 4-aminoquinoline and a 1,2,4-trioxane, has been reported to possess greater broad-spectrum schistosomicidal activity against all life stages of *S. mansoni* [21, 26].

**Fig. 1:**
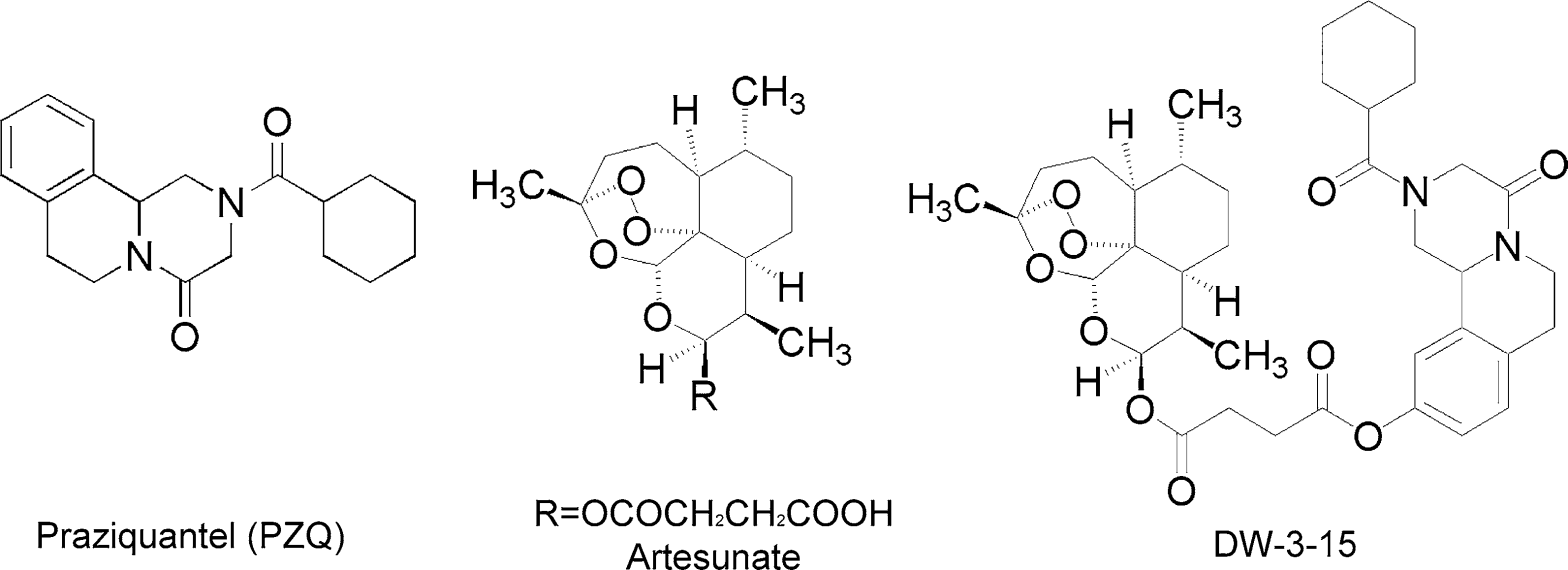
Chemical structures of praziquantel (PZQ), artesunate and DW-3-15

In view of the complementary activities of PZQ and artemisinin against schistosomes, DW-3-15 (patent NO: ZL201110142538.2), a hybrid compound linked to the endoperoxide bridge of artemisinin at position 10 of PZQ, was developed in our laboratory (Fig. 1). In previous studies, DW-3-15 was shown to be effective against all developmental stages. DW-3-15 exhibited dual action *in vivo*, namely activity against juvenile worms comparable to that of artesunate, and a 60–85% reduction in total worm burden at different stages of *S. japonicum* development. Notably, DW-3-15 reduced the total worm burden by 75–85.7% on day 1 and 3 post-infection, whereas both artesunate and PZQ were only slightly active [27]. If a new compound shows a potential for use in treating shistosomiasis during preclinical and clinical trials, it is essential that the compound can be synthesized in bulk through commercial channels. In the present study, synthesis of DW-3-15 was performed by a pharmaceutical company and the schistosomicidal efficacy and stability of the compound were further confirmed.

The effects of commercial DW-3-15 on the viability and oviposition of adult and juvenile *S. japonicum* worms were assessed *in vitro*. Moreover, the antiparasitic activity of commercial DW-3-15 in a mouse model of *S. japonicum* infection was investigated, using PZQ as a positive control. Worm burden, egg burden and diameter of granuloma in the liver were evaluated in all the treatment groups. In addition, to elucidate the potential mechanisms of the compound’s antischistosomal action, ultrastructural alterations in DW-3-15-treated male *S. japonicum* parasites were also examined.

## Methods

### Chemistry and reagents

Praziquantel derivative DW-3-15 (Fig. 1) was prepared by WuXi App Tec Co. Ltd (Shanghai, China). The synthetic route and chemically characterized data of DW-3-15 are described in Additional file 1. Praziquantel (PZQ), glutaraldehyde solution and formalin were purchased from Sigma Aldrich (St. Louis, MO, USA). Dulbecco’s modified minimum Eagle’s medium (DMEM) and penicillin/streptomycin were purchased from Life Technologies (Carlsbad, CA, USA). All compounds for the *in vitro* assay were dissolved in dimethylsulfoxide (DMSO, Fluka, Buchs, Switzerland). Newborn calf serum was purchased from Biological Industries (Cromwell, CT, USA). Paraffin was supplied by Merck (Darmstadt, Germany).

### Parasites and animals

*Schistosoma japonicum* cercariae (Chinese mainland strain) used throughout this study emerged from the infected intermediate host snail *Oncomelania hupensis*, which were obtained from Shanghai Municipal Center for Disease Control and Prevention (Shanghai, China). Female ICR mice (4–6 weeks-old, approximate weight: 20 g) provided by the Experimental Animal Center of Soochow University (Suzhou, China), were used to study the mammalian stages of the *S. japonicum* life-cycle. The animals were maintained under a controlled temperature (20 ± 2 °C) and photoperiod (12 h light, 12 h dark), and had access to water and food *ad libitum*. Mice were acclimated to these conditions before being infected. Cercariae were obtained from infected snails exposed to light for approximately 1 h. The mice were individually infected transcutaneously with approximately 60 *S. japonicum* cercariae.

### *In vitro* studies

For the *in vitro* bioassay, worms recovered from infected mice at 16 days (juvenile worms) and 35 days (adult worms) post-infection were collected through perfusion of the hepatic portal system and mesenteric veins using citrated saline according to the technique reported by Duvall et al. [28]. Harvested *S. japonicum* specimens were maintained in complete DMEM supplemented with 10% heat-inactivated newborn calf serum, 100 U/ml of penicillin and 100 μg/ml streptomycin. The worms were placed in 6-well plates (Corning Costar, Corning, New York, USA), five worms per well, and separated by sex. These cultures were stored at 37 °C in an atmosphere of 5% CO_2_. The final concentrations of DW-3-15 were 25, 50, 75 and 100 μM. The negative control group was incubated with complete DMEM containing 0.1% DMSO, whereas the positive control group was incubated with 100 μM PZQ. Adult and juvenile *S. japonicum* worms were exposed to the drugs for 24 h, rinsed three times with DMEM the next day, and subsequently cultured in drug-free complete DMEM for 72 h. The worms were observed at 24, 48, 72 and 96 h post-incubation under a dissecting microscope (MZ 12.5, Leica, Wetzlar, Germany) and assigned a viability score based on motor activity and morphological changes.

The phenotypic changes were scored using a viability scale of 0–3 [29]: 0, parasites did not resume movements for 1 min, remained contracted, deemed as dead; 1, parasites moved partially and had an opaque appearance; 2, parasites had full-body movement but moved stiffly and slowly, and had a translucent body; 3, this was the state observed in the control group during the whole observation period, worms moved actively and normally, and had a transparent body. The formula of the viability score for each sample was used as previously described [30]. Briefly, the viability score = ∑(worm scores)/number of worms. All measurements were taken in triplicate and each experiment was repeated at least three times. IC_50_ values were calculated from viability values at different concentrations of DW-3-15 using GraphPad software (version 5.0).

### Worm pairing and egg output studies *in vitro*

One pair of adult worms was transferred to each well of a 12-well culture plate (Corning) containing complete DMEM and different concentrations of DW-3-15 (25–100 μM) as described above. During the culture period, the pairs were examined by visual inspection using an inverted microscope (Olympus CKX41, Tokyo, Japan) at 1, 6, 12 and 24 h. Moreover, eggs laid in the well were collected and counted immediately after 24 and 96 h of incubation with DW-3-15. In the negative control, pairs of adult worms and eggs were incubated with complete DMEM containing 0.1% DMSO. The percentage pairing was expressed as pairing worms relative to the negative control group. The experiments were carried out in quintuplicate and repeated three times.

### Scanning electron microscopy

After 96 h of exposure to 100 μM of DW-3-15 and PZQ *in vitro*, samples of male worms were fixed overnight at 4 °C in 2.5% glutaraldehyde-PBS buffer solution (pH 7.4), washed three times in PBS buffer, and then fixed again in 1% osmium tetroxide (OsO_4_) for 1 h. Next, the samples were dehydrated in an ascending ethanol series (30, 50, 70 and 90%) for 10 min in each solution, then washed in 100% ethanol. Finally, the samples were placed in a drier for approximately 30 min, mounted on aluminum stubs, sputtered with a coat of gold, and examined under a Hitachi-S4700 scanning electron microscope (Chiyoda-ku, Japan).

### *In vivo* studies

DW-3-15 in 200 μl of ethanol:Tween 80:water (3:7:90) solvent mixture was administered at a dose of 400 mg/kg per day for 5 days by gavage to groups of five mice, 35 days after *S. japonicum* infection. The negative control consisted of five infected mice receiving 200 μl of the ethanol:Tween 80:water (3:7:90) solvent mixture, and the positive control received PZQ at 400 mg/kg per day for 5 days. After five consecutive days of treatment, all mice were euthanized with an overdose of sodium pentobarbital (200 mg/kg) and *S. japonicum* worms were collected from all groups by perfusion.

To evaluate egg burden, half of the livers were homogenized in a 1.2% NaCl solution. Next, the sedimentary deposits were collected and centrifuged at 1500×*g* for 20 min on Percoll with a density of 1.070 [31]. The eggs in the pellet were observed under a microscope at 100× magnification. Percent reduction in worm burden and number of eggs was calculated as follows: % reduction = [(value of control group − value of treatment group)/value of control group] × 100.

For histological analysis, the other half of the livers from all groups were fixed in 4% neutral buffered formalin, dehydrated in increasing concentrations of ethanol, diaphonized in xylol and then embedded in paraffin. Sections were dewaxed and stained with H&E for granuloma analysis. The diameters of egg granuloma in each group were calculated as previously described [19].

### Statistical analysis

All statistical analyses were performed using GraphPad Prism software (version 5.0). All data were expressed as the mean value ± standard deviation (SD). Differences between groups were analyzed by one-way ANOVA followed by Dunnett’s test and the Kruskal–Wallis (K–W) test. A *P*-value < 0.05 or less was considered statistically significant.

## Results

### DW-3-15 alters the viability of adult and juvenile worms *in vitro*

**Fig. 2:**
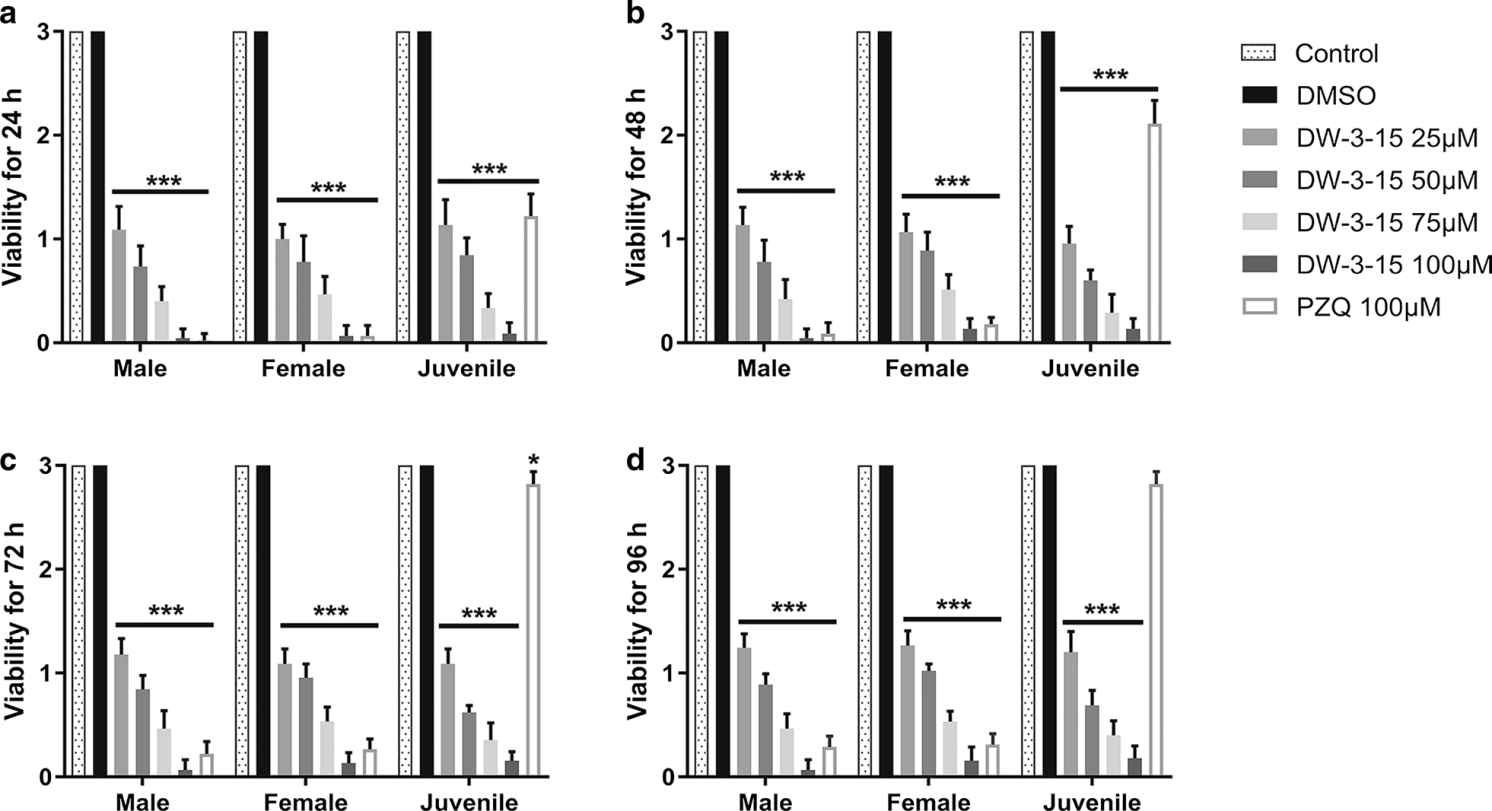
DW-3-15 altered the viability of *S. japonicum* adult and juvenile worm *in vitro*. Male, female, and juvenile worms were incubated with DW-3-15 or PZQ at different concentrations for 24 (**a**), 48 (**b**), 72 (**c**) and 96 h (**d**). Adult and juvenile worms were exposed to the drugs for 24 h, rinsed three times with DMEM the next day, and subsequently cultured in drug-free complete DMEM for 48, 72 and 96 h. The viability was evaluated using a viability score of 0–3. The negative control group was incubated with complete DMEM with 0.1% DMSO, and complete DMEM alone was used for the control group. Significant differences compared to the negative control group are indicated by *P < 0.05, **P < 0.01 and ***P < 0.001

DW-3-15 was tested at concentrations of 2.5, 5 and 10 μM, but no significant worm mortality was observed in the pre-experiment (data not shown). Therefore, DW-3-15 was used at concentrations greater than 25 μM. Worm viability was evaluated daily by optical observation as described in the Methods section. Throughout the incubation period, both adult and juvenile worms in the negative control group remained active and moved normally. As expected, PZQ was effective against adult worms and mildly effective against juvenile worms. Notably, DW-3-15 showed a pronounced lethal effect against both adult and juvenile *S. japonicum* worms. As shown in Fig. 2, higher concentrations of DW-3-15 resulted in significantly reduced viability in male, female and juvenile stages at 24, 48, 72 and 96 h.

There was a significant reduction in the viability score of both male (24 h: *F*_(6,56)_ = 857.4, *P* < 0.001; 48 h: *F*_(6,56)_ = 808.3, *P* < 0.001; 72 h: *F*_(6,56)_ = 1017, *P* < 0.001; 96 h: *F*_(6,56)_ = 1378, *P* < 0.001) and female worms (24 h: *F*_(6,56)_ = 765.8, *P* < 0.001; 48 h: *F*_(6,56)_ = 1003, *P* < 0.001; 72 h: *F*_(6,56)_ = 1192, *P* < 0.001; 96 h: *F*_(6,56)_ = 1447, *P* < 0.001) treated with 25–100 μM DW-3-15. The lethal effect of DW-3-15 was concentration-dependent, with DW-3-15 at 100 μM showing the most potent effect. We also examined mortality in adult worms and found that nearly all deaths occurred within 24 h after treatment with 100 μM DW-3-15 (Fig. 2a). In contrast, DW-3-15 at concentrations of 25, 50 and 75 μM elicited a modest reduction in viability. Following exposure for 24 h, DW-3-15 (25–100 μM) exerted antiparasitic activity against males and females *in vitro*, with IC_50_s of 20.36 and 20.8 μM, respectively.

After 24 h of DW-3-15 exposure, the worms were washed and incubated in drug-free medium. There were no significant differences in the viability scores at the same concentrations of DW-3-15 between the 48, 72 and 96 h incubation periods. The majority of worms shortened with rod-type body shape presented a complete loss of movement and sucking capacity; they became opaque or white, and were characterized as ‘dead’. Conversely, after an overnight exposure to PZQ, male and female worms showed some signs of recovery 96 h after treatment; from 24 h to 96 h incubation period, PZQ reduced the viability scores of males and females by 90.4–99.3% and 89.6–97.8%, respectively. The viability scores of male and female worms incubated with DW-3-15 at 100 μM were lower than for the worms in the PZQ group (Fig. 2b–d).

Under the same experimental conditions, a significant reduction in the viability score of juvenile worms treated with 25–100 μM DW-3-15 was also observed (24 h: *F*_(6,56)_ = 538.9, *P* < 0.001; 48 h: *F*_(6,56)_ = 750.4, *P* < 0.001; 72 h: *F*_(6,56)_ = 1426, *P* < 0.001; 96 h: *F*_(6,56)_ = 941.7, *P* < 0.001). Exposure to 100 μM DW-3-15 resulted in almost 100% reduction in the viability of juvenile parasites within 24 h, and the IC_50_ value was 21.22 μM. There was no significant difference in the viability score at the same concentration of DW-3-15 for the 48, 72 and 96 h incubation periods (Fig. 2). However, juvenile worms placed in drug-free medium after 24 h exposure to PZQ presented evident signs of recovery 96 h post-treatment, as previously reported [32].

### DW-3-15 inhibits worm pairing and egg output *in vitro*

**Fig. 3:**
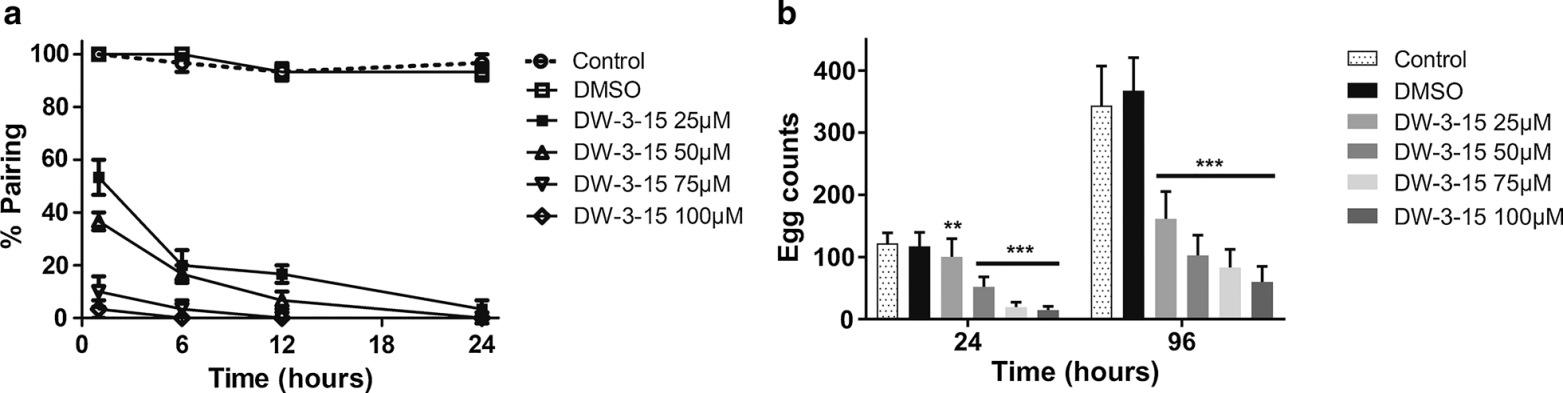
DW-3-15 inhibited worm pairing and egg output *in vitro*. Adult couples were incubated with the indicated concentrations of DW-3-15 and worm pairing and total egg counts assessed as described in methods. **a** Pairing curves of adult couples for 24 h; **b** total egg counts normalized to worm couples for 24 and 96 h. The negative control group was incubated with complete DMEM containing 0.1% DMSO. The control group was incubated with complete DMEM alone. Significant differences compared to the negative control group are indicated by **P < 0.01 and ***P < 0.001

To evaluate the inhibitory effect of DW-3-15 on worm pairing and egg production, 35-day-old worm couples were treated with 25–100 μM DW-3-15. During the assay, worm couples in the negative control group moved actively and remained paired. Similar to the results shown in Fig. 2, 25–100 μM DW-3-15 strongly reduced the viability of the pairs. After treatment with 25 and 50 μM DW-3-15, approximately 50% of the worms stayed paired, whereas exposure to 75 or 100 μM DW-3-15 resulted in nearly 100% separation of worm couples within 1 h of incubation. All adult worm pairs separated within 24 h of incubation with DW-3-15 at 25–100 μM (Fig. 3a), and the worms remained separated over the next cultivation periods. Therefore, DW-3-15 exhibited an inhibitory effect on natural mating, resulting in the separation of worm couples *in vitro*.

Egg output of adult worm pairs cultured with different concentrations of DW-3-15 for 24 or 96 h was significantly lower than for the negative control (24 h: *F*_(5,84)_ = 105.5, *P* < 0.001; 96h: *F*_(5,84)_ = 144.9, *P* < 0.001, Fig. 3b). Reduced egg production may be attributed both to the DW-3-15-induced separation of adult worm pairs and a decrease in worm viability.

### DW-3-15 induces severe damage to the integument of male worms observed *in vitro*

**Fig. 4:**
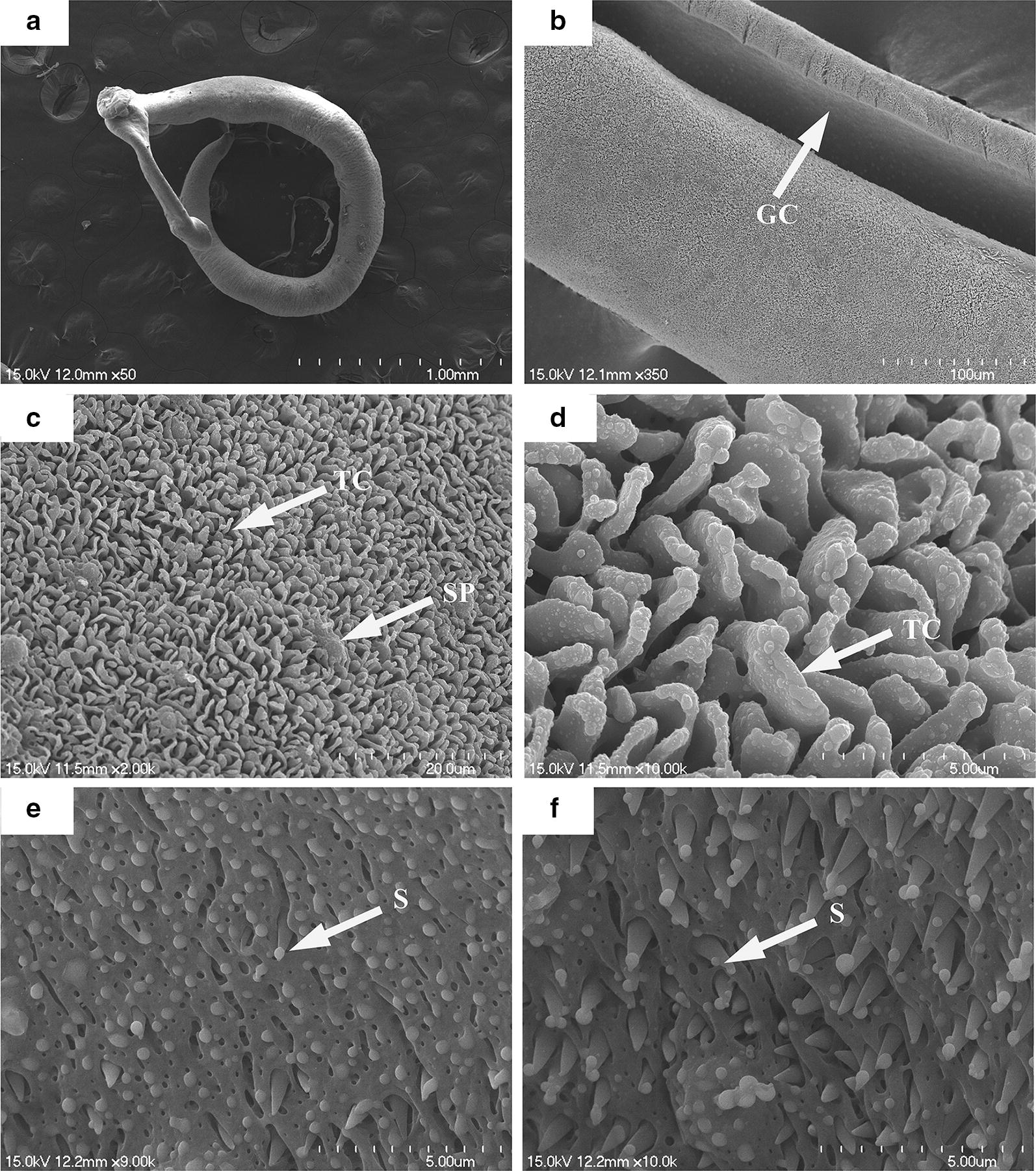
Scanning electron micrographs of *S. japonicum* male worms from the negative control group. Worms after 96 h of incubation showed no abnormalities in tegument topography (**a**) and the gynecophoral canal (GC) (**b**). In detail, numerous tegumental crests (TC) with sensory papillae (SP) were distributed along the body (**c**, **d**) and the inner wall of the GC showed high integrity and prominent spines (S) (**e**, **f**). *Scale-bars*: **a**, 1000 µm; **b**, 100 µm; **c**, 20 µm; **d**–**f**, 5 µm

Under scanning electron microscopy, we observed that the control male worms showed normal tegument topography (Fig. 4a, b). The distribution and size of crests and sensory papillae were consistent along their body (Fig. 4c, d). In addition, no morphological alteration was observed in the gynecophoral canal, and the inner wall and typical spines were preserved (Fig. 4e, f).

**Fig. 5:**
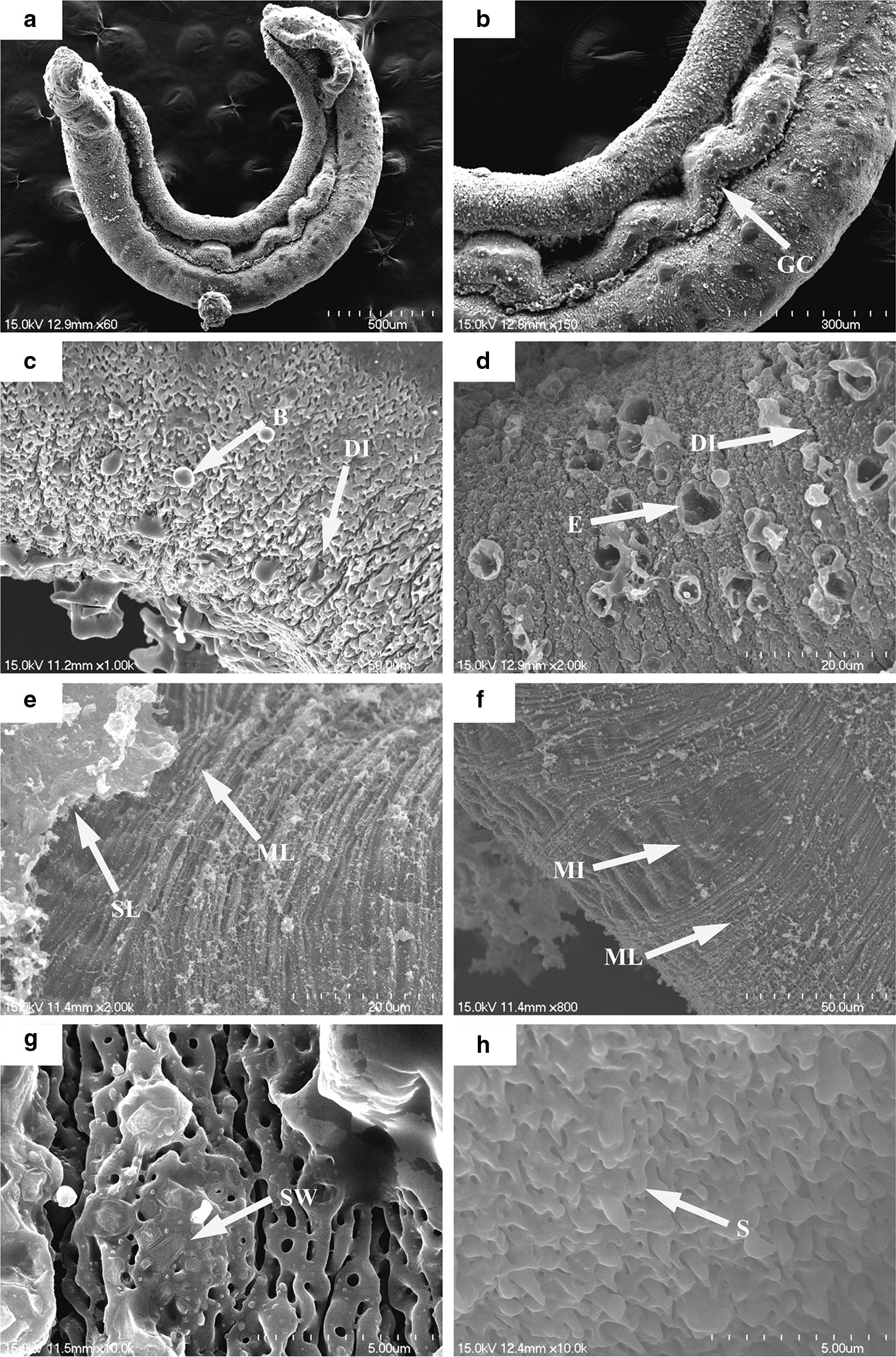
Scanning electron micrographs of *S. japonicum* male worms exposed to DW-3-15. After 96 h of incubation, worms became shortened (**a**) and showed severe damage to the gynecophoral canal (GC) (**b**), disintegration (DI) of the tegumental crest, blisters (B), hole-shaped erosion (E), extensive sloughing (SL) with exposure of the subtegumental muscle layer (ML) and muscle injury (MI), and swelling (SW) (**c**–**g**) and the inner wall of the gynecophoral canal showed surface fusion with spine loss (S) (**h**). *Scale-bars*: **a**, 500 µm; **b**, 300 µm; **c**, 50 µm; **d**, **e**, 20 µm; **f**, 50 µm; **g**, 5 µm; **h**, 5 µm

In contrast, the males exposed to DW-3-15 showed ultrastructural alterations, with significant changes in the tegument (Fig. 5a, b). In male worms, blisters and hole-shaped erosions were observed, as well as destruction of crests and sensory papillae, which showed the disintegration and shortening of such structures in various areas along the body (Fig. 5c, d); swelling and extensive sloughing of the tegument were also observed, with exposure of the sub-tegument layer of muscle tissue and even injury to the muscle tissue layer (Fig. 5e–g). The gynecophoral canal of males was seriously damaged, showing fusion of the inner wall and loss of spine (Fig. 5h).

**Fig. 6:**
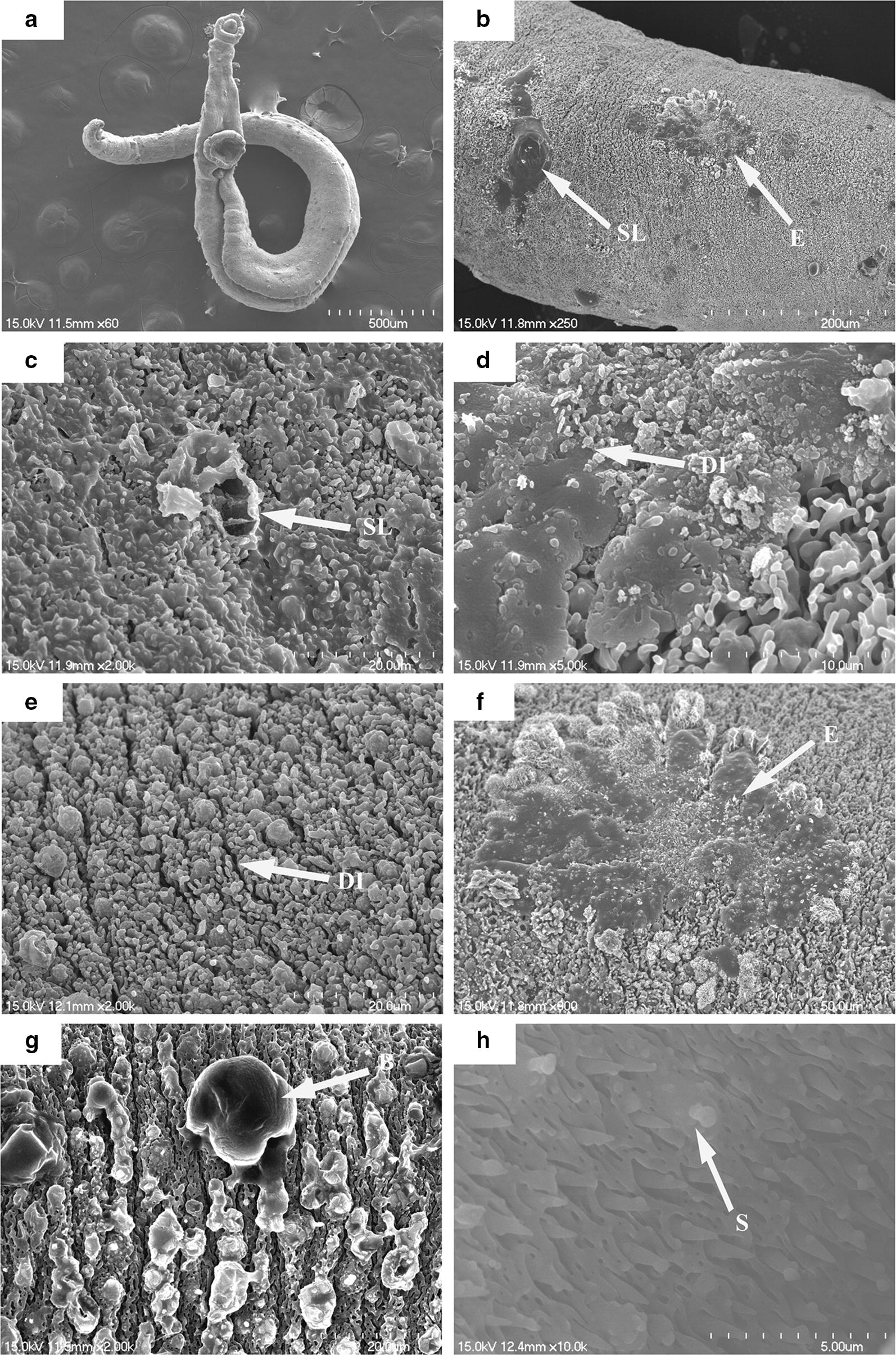
Scanning electron micrographs of *S. japonicum* male worms exposed to PZQ. Worms after 96 h of incubation were curled up (**a**), and showed shallow sloughing (SL) (**b**, **c**), disintegration (DI) of the tegumental crest, pit-shaped erosion (E) and blisters (B) (**d**–**g**). In the gynecophoral canal, disarrangement of the inner wall and loss of spine (S) were visible (**h**). *Scale-bars*: **a**, 500 µm; **b**, 200 µm; **c**, **e**, **g**, 20 µm; **d**, 10 µm; **f**, 50 µm; **h**, 5 µm

Male worms exposed to 100 μM PZQ showed various changes in the tegument, with destruction of crests, shallow peeling, and pit-shaped erosion. Disintegration of the tegument and formation of blisters were observed around the crests (Fig. 6a–g). In the gynecophoral canal, disarrangement of the inner wall and loss of spines were observed (Fig. 6h). The changes were confined to the tegument surface of the worm, with no damage observed in the muscle tissue layer.

### DW-3-15 reduces worm burden and egg formation *in vivo*

After observing the potent antischistosomal activity of DW-3-15 *in vitro*, we further evaluated its effect on adult worms *in vivo*. There were statistically significant differences in the total worm burden (K–W test, *H* = 12.64, *P* < 0.01) and number of eggs (K-W test, *H* = 12.50, *P* < 0.01) between treated and control groups, as shown in Table 1. Treatment with DW-3-15 resulted in a 63.4% reduction in total worm burden. The reduction in the female worm burden (70.8%) was higher than the reduction in the male worm burden (57.2%). Additionally, DW-3-15 exposure led to significant reduction (85.8%) in the number of eggs. Treatment with PZQ at 400 mg/kg resulted in 98.9, 100 and 97.9% reductions in total worm, female and male burden, respectively, and a 97.0% reduction in egg numbers. These reductions were higher than those induced by the DW-3-15 treatment.

**Table 1:** *In vivo* effect of DW-3-15 against *S. japonicum* infected mice

Group and dose (mg/kg per day)^a^	Worm burden (Mean ± SD)			Worm burden reduction (%)			No. of eggs × 10^3^/g liver tissue	Egg reduction (%)
	Female	Male	Total	Female	Male	Total		
Control^b^	24.0 ± 1.9	29.0 ± 2.3	53.0 ± 3.4	–	–	–	23.8 ± 5.2	–
DW-3-15(400)	7.0 ± 2.0	12.4 ± 1.8	19.4 ± 1.7*	70.8	57.2	63.4	3.4 ± 0.5*	85.8
PZQ(400)	0 ± 0	0.6 ± 0.5	0.6 ± 0.5*	100.00	97.9	98.9	0.7 ± 0.3*	97.0

^a^Treatment was started 35 days after infection

^b^Control, 200 μl of ethanol:Tween80:water (3:7:90)

*P < 0.05, significant difference compared to the control group

*Abbreviation*: SD, standard deviation

**Fig. 7:**
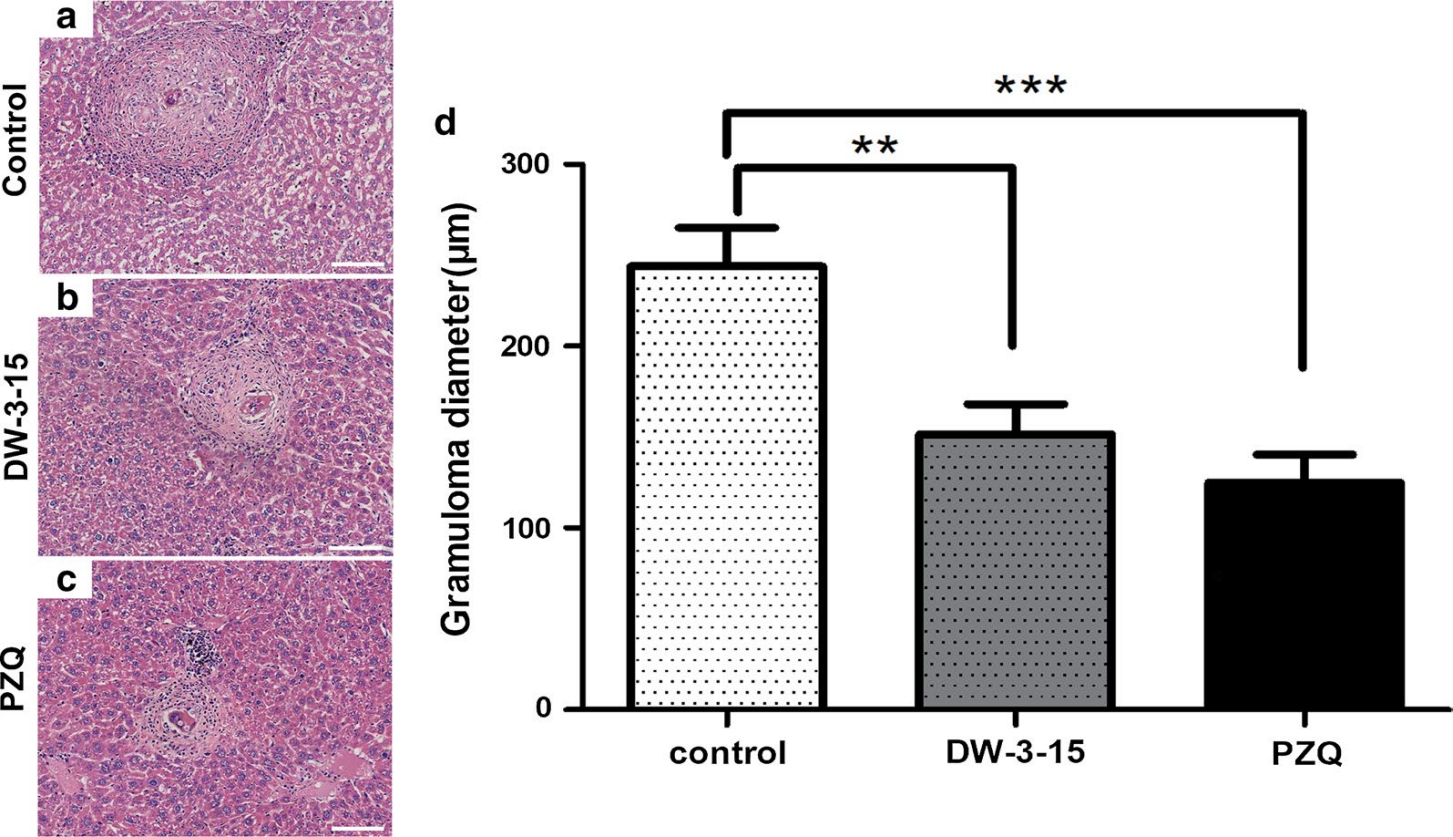
*In vivo* effect of DW-3-15 on hepatic granulomas in *S. japonicum*-infected mice. Photomicrograph of hepatic granulomas in mice of the control group (**a**), mice treated with DW-3-15 400 mg/kg per day (**b**) and mice treated with PZQ 400 mg/kg per day (**c**). **d** Significant differences compared to the control group are indicated by **P < 0.01 and ***P < 0.001. *Scale-bars*: **a**–**c**, 100 μm

Microscopical examination of the livers from untreated infected mice revealed granulomatous lesions formed by numerous eggs surrounded by inflammatory cells. After treatment with DW-3-15, the granuloma showed a marked decrease in diameter, fewer eggs, and moderate inflammatory-cell infiltration (Fig. 7). Histological analysis showed that the average granuloma diameter was significantly smaller in the groups treated with DW-3-15 or PZQ than in the untreated control group (*F*_(2,147)_ = 12.83, *P* < 0.001). There was no significant difference in the average diameter of hepatic granuloma in DW-3-15-treated mice compared to the positive control group.

## Discussion

Without available alternative drugs, PZQ, a veterinary medicine repurposed for human use, is currently the drug of first choice recommended for treating and controlling schistosomiasis [33, 34]. The WHO has provided opportunities for the development of alternative antischistosomal drugs for such a serious disease, and although several promising compounds have been shown to kill *Schistosoma*, their efficacy is not equivalent to that of PZQ [19, 21]. Moreover, the efficacy of PZQ depends on the developmental stage of the worms and, in *in vitro* studies, on whether the infection was bisexual or unisexual [32]. Apart from its unclear mechanism of action, an additional drawback of PZQ is the potential for drug resistance or tolerance. A desirable therapeutic profile for candidate drugs includes not only the ability to kill both juvenile and adult worms, but also efficacy against schistosomula. Considering the complementary effects of PZQ and artemisinin on schistosomes [11, 23, 25], novel hybrid molecules with multiple modes of action that fulfill these therapeutic profiles are attractive options for drug development and testing.

In our previous studies, we synthesized DW-3-15 by linkage of artesunate to PZQ through the introduction of a hydroxyl group at the 10-position of PZQ, and observed its potent effect in reducing total worm burden at all developmental stages of *S. japonicum* (day 1 to 35 post-infection) [27]. In this study, our results provide further evidence that patent DW-3-15, a PZQ derivative with potential for commercial production, is not only a potent active compound superior to PZQ against adult and juvenile worms *in vitro,* but also exhibits promising antischistosomal properties *in vivo*. In addition, DW-3-15 induces significant morphological alternations in the tegument of adult worms, similar to PZQ.

Our *in vitro* data showed that the lethal effect (decreased viability) of DW-3-15 was dose-dependent, and the most effective dose was 100 μM. Male worms seemed more sensitive to DW-3-15 than female worms of the same age (Fig. 2). A similar sensitivity of *S. mansoni* to PZQ was observed by Pica et al. [32]. Furthermore, DW-3-15 exhibited inhibitory effects on worm pairing and egg production, which were both dose- and time-dependent. Significantly, when tested at sub-lethal concentrations, DW-3-15 also reduced egg output (Fig. 3). A unique characteristic of schistosome biology is that differentiation and maturation of female reproductive organs are highly dependent on the male. Indeed, worm pairing is considered essential to induce oviposition [35, 36]. DW-3-15 could potentially inhibit, directly or indirectly, worm pairing and oviposition, interfering with essential biological process and thus controlling schistosomiasis.

The consistently high reduction in the viability of adult and juvenile stages *in vitro* of *S. japonicum* seems to be an advantage of DW-3-15 compared to PZQ. It is well known that PZQ exerts high activity against adult worms and reduced activity against immature worms (2- to 3-week-old) [37]. The *in vitro* results showed that juvenile worms exposed to PZQ presented evident signs of recovery and adult worms presented mild recovery 96 h post-treatment, which may explain the reported treatment ‘failures’ in schistosomiasis-endemic areas. In the murine model of schistosomiasis, the dose of DW-3-15 is normally selected based on doses used in previous studies [27], and PZQ is used at the near-curative oral dose [34]. The *in vivo* effect of DW-3-15 against female worms was superior to that against male worms, and also caused a 63.4% reduction in total worm burden. Although DW-3-15 exhibits slightly higher activity than PZQ *in vitro*, it does not reduce worm burden nearly as well as PZQ, and the treatment is administered over five days. This might be due to different modes of action, or the possible binding of DW-3-15 molecules to different active sites *in vivo*, and further dose-response tests (with both drugs) would be needed for a meaningful comparison. Because of the limited aqueous solubility of DW-3-15, formulations should be developed to promote greater biological activity and bioavailability, and determine the appropriate administration route and optimal dosage.

Tegument integrity is essential for a *Schistosoma* worm. This structure is involved in a variety of physiological processes like nutrient absorption, proliferation and energy metabolism, and additionally also expresses proteins that help to evade the host immune system. The parasite interacts with the host environment *via* the thick tegument, which is its first protective barrier. This renders the tegument a potential target for numerous drugs, and alterations to the tegument could also be used to evaluate the antischistosomal activity of a drug [29, 38, 39]. For instance, Xiao et al. [40] reported contraction and swelling of the tegument, fusion and blisters of the tubercles, and disorder of the spines in *S. mansoni* exposed to artemether. Moreover, Santiago et al. [29] reported LpQM-45-induced changes in the tegument surface of adult *S. mansoni* worms, including extensive peeling, exposure of the subtegumental muscle layer and loss of tubercles.

Scanning electron microscopy was used to qualify changes in the tegument topography. The clear damage observed in male worms treated with DW-3-15 was similar to treatment with PZQ, like the destruction of the crests, extensive peeling with disintegration of the tegument, swelling and exposure of the subtegumental layer and/or muscle tissue (Figs. 5, 6). Similar morphological changes were observed in the tegument of *S. japonicum* exposed to DW-3-15, including roughened tegument with a disordered surface, as reported in the *in vivo* studies [27]. Our results also showed that the cell membranes and lipid bilayers of *S. japonicum* worms were extremely vulnerable to DW-3-15. Morphological changes induced by DW-3-15 may induce profound effects upon the metabolic function of the parasite and may be the mechanism responsible for the efficacy of DW-3-15 against schistosomes. Damage to the tegument surface and along the body may destroy the integrity of the tegument and impair the worm’s defense system, causing it to be easily overwhelmed by the host’s immune system through surface antigen exposure [21]. These differential degenerative changes in the tegument might be helpful in elucidating the mechanisms of action of PZQ and DW-3-15.

In schistosomiasis japonica, granulomatous lesions around the eggs lead to chronic liver inflammation and fibrosis due to excessive deposition of collagen in the extracellular matrix. Egg-induced granuloma is considered the primary pathogenic agent of schistosomiasis. Our results showed that DW-3-15 induced significant reductions in egg burden *in vivo* and egg production *in vitro*. In addition, there was a significant reduction in the average diameter of hepatic granuloma in mice treated with DW-3-15, indicating the anti-inflammatory effect of DW-3-15. Similar results have been reported for other compounds, including 8-hydroxyquinoline derivatives and pomegranate extract [41, 42]. Earlier studies reported that the reduction in granuloma diameter was significantly related to egg burden eliminated in the feces [43]. In the present study, DW-3-15 treatment significantly attenuated the egg burden in the liver, and reduced the diameter of egg-induced granuloma. These findings strongly suggest that DW-3-15 not only possesses antiparasitic activity, but also inhibits the formation of egg-induced granulomas, indicative of a hepatoprotective role against schistosomiasis japonica.

The pharmacophore of DW-3-15 originates partly from artemisinin derivatives. Artemisinin derivatives are characterized by their anti-malarial activity but some, like mefloquine [44] and artesunate [45], are reported to also possess antischistosomal activity. Since artemisinin derivatives are vital for the treatment of malaria, the malaria community has argued that these drugs should therefore not be used against schistosomiasis because of the risk that this therapy may select for resistant *Plasmodium* parasites in co-endemic areas. However, many malaria patients co-infected with schistosomes either have been, or will be, treated with artemisinin-based combination therapies (ACTs) [44]. Hybrid analogues based on conjugation of targeted pharmacophores may circumvent this problem. Our previous and current data also provide a good proof of concept that the strategy of using chimeric drugs is feasible, suggesting the use of DW-3-15 as a prototype for the development of new analogues with potential schistosomicidal properties. Considering the potent activity of DW-3-15 against *S. japonicum*, further pharmacokinetics and structure-activity relationship studies are needed to elucidate the mechanism of action.

## Conclusions

Our results showed that DW-3-15, a commercialized PZQ derivative, exerted schistosomicidal effect on adult and juvenile *S. japonicum*, including a significant reduction in worm viability, oviposition, worm burden and number of eggs in the liver. Notably, DW-3-15 induced clear damage to the tegument of adult worms similar to the reference drug (PZQ), and inhibited the formation of egg-induced granuloma in the liver. The present work strongly encourages the development of DW-3-15 as a potential promising candidate drug for treating schistosomiasis *japonica*. Further in-depth studies are needed to fully elucidate the mechanism of antischistosomal action of DW-3-15, and to address the suitable dosage regimen for treating schistosomal infection in humans.

## Additional file

**Additional file 1.** The synthesis and chemical characterization data of DW-3-15.

## Abbreviations

PZQ: praziquantel
WHO: World Health Organization
DALYs: disability-adjusted life years
DMSO: dimethyl sulfoxide
IC_50_: concentration of the drug needed to kill 50% of the parasites
ACTs: artemisinin-based combination therapies
