# Supplementary material for: In vitro and in vivo activities of DW-3-15, a commercial praziquantel derivative, against Schistosoma japonicum
Source: Parasit Vectors. 2019 May 3;12:199. doi: 10.1186/s13071-019-3442-7 (PMC6500042; doi:10.1186/s13071-019-3442-7)
Supplement: Supplementary file 1 — Additional file 1. The synthesis and chemical characterization data of DW-3-15. [file 13071_2019_3442_MOESM1_ESM.docx]

### WuXi Apptec Co., Ltd.

**Final Report to SUDA**

**Synthesis of SUDA-20170906**

**Delivery Summary**

A request was made by **SUDA-20170906** on **Oct_10_2017** to synthesize 10 g of **SUDA-20170906** with a delivery date on **Nov_1_2017**.

| **Compound ID** | **Amount Delivered (g/mg)** | **Date Delivered** | **#Step** | **Notebook Page** |
| --- | --- | --- | --- | --- |
| **Dw-3-15** | **10.15 g** | **Nov. 1, 2017** | **4** | **ES8620-5** |

**Date Started: Oct_10_2017**

**Date Completed: Nov_1_2017**

**WX Chemist: Qifeng Zhou**

# **Project Summary**

The synthetic route is based on a procedure supplied by **SUDA-20170906**

##### Experimental for largest scale run:

***General procedure for preparation of compound* *2*** *-* ***Notebook Page: ES8620-1***

To a solution of ***compound 1*** (200 g, 640.19 mmol, 1 *eq*) in H_2_SO_4_ (2000 mL) was added HNO_3_ (124.12 g, 1.28 mol, 88.66 mL, 65% purity, 2 *eq*)    in dropwise at 0 °C,after the addition completed, then the reaction mixture was stirred for 3 hr at 0 °C.  LC/MS (ES8620-1-P1A) showed the ES8620-1-R1 was all consumed completely, and mainly ES8620-1-P1 (R.T. = 0.771 min, [M+H] = 358.1). The reaction mixture was poured into 8 L ice water in portions, then DCM (2.5 L x 3) was added to extract the product. The combined organic layer was dried over Na_2_SO_4_ and concentrated to give 220 g crude yellow gum. The crude product was purified by silica gel column chromatography (PE / EA = 3 : 1 ~ 1 : 2 ). Then the fraction was concentrated in vacuum to give 150 g yellow gum. ^1^H-NMR (ES8620-1-P1N1) and NOE (ES8620-1-P1N2) showed the ***compound 2*** (150 g, 419.69 mmol, 65.56% yield) was obtained as a yellow gum.

LCMS: ES8620-1-P1A ^1^H NMR: ES8620-1-P1N1 NOE: ES8620-1-P1N2

***General procedure for preparation of compound* *3*** *-* ***Notebook Page: ES8620-2***

To a solution of ***compound 2*** (120 g, 335.76 mmol, 1 *eq*) and Raney-Ni (12 g, 140.06 mmol, 4.17e-1 *eq*)  in MeOH (1200 mL)  was added N_2_H_4_.H_2_O (34.30 g, 671.51 mmol, 33.30 mL, 98% purity, 2 *eq*) in dropwise at 0 °C.  After the addition completed, then the mixture was stirred for 16 h at 20 °C.  LC / MS (ES8620-2-P1A) showed the ES8620-2-R1 was all consumed completely, and mainly ES8620-2-P11-P1 (R.T. = 0.658 min, [M+H] = 328.3). Filter to remove Raney-Ni (12 g, 140.06 mmol, 4.17e-1 *eq*), then concentrate to remove MeOH (1200 mL). 1 L NH_4_Cl was added into the mixture and DCM (1 L x 2) was added to extract the product. The organic layer was concentrated to give 125 g light yellow solid.  ^1^H-NMR (ES8620-2-P1N1) showed the ***compound 3*** (125 g, crude) was obtained as a light yellow solid.

LCMS: ES8620-2-P1A ^1^H NMR: ES8620-2-P1N1

***General procedure for preparation of compound* *4*** *-* ***Notebook Page: ES8620-3, ES8620-4***

To a solution of ***compound 3*** (30 g, 91.63 mmol, 1 *eq*) in H_2_SO_4_ (300 mL, 5% purity)   was added  NaNO_2_ (6.95 g, 100.79 mmol, 1.1 *eq*)   (solution in 60 mL water) dropwise at 0 °C, then the mixture was stirred for 0.5 hr at 0 °C. Meanwhile, H_2_SO_4_ (1000 mL, 20% purity)   was prepared and stirring with reflux at 150 °C. The reaction mixture was added to the 20% H_2_SO_4_ solution dropwise. After the addition completed, then the mixture was stirred at 150°C for 1 h. LC / MS (ES8620-3-P1A) showed ***compound 3*** was all consumed, and mainly ***compound 4*** (R.T. = 0.724 min,  [M+H] = 329.1). The reaction mixture was cooled down to 30 °C, then added EtOAc (500mL x 3) to extract. The organic layer was concentrated to give 15 g yellow solid. The residue was slurried in a solution of PE / EA (v / v = 1 / 1, 50 mL) at 25 °C for 15 min. The mixture was then filtered, and the residue was dried in vacuum to give 10 g of yellow solid. LC / MS (ES8620-3-P1L2) and ^1^H-NMR (ES8620-3-P1N2) showed ***compound 4*** (10 g, 30.45 mmol, 33.23% yield) was obtained as a yellow solid.

LCMS: ES8620-3-P1A LCMS: ES8620-3-P1L2 ^1^H NMR: ES8620-3-P1N2

***General procedure for preparation of Dw-3-15*** *-* ***Notebook Page: ES8620-5***

To a solution of ***compound 4*** (5 g, 15.23 mmol, 1 *eq*) and ***compound 5A*** (8.78 g, 22.84 mmol, 1.5 *eq*) in DCM (50 mL) was added HOBt (2.16 g, 15.99 mmol, 1.05 *eq*) at 0 °C. Then EDCI (3.06 g, 15.99 mmol, 1.05 *eq*) was added in portions at 0 °C. Then DMAP (186.00 mg, 1.52 mmol, 0.10 *eq*) was added in one portion at 0 °C. After the addition completed, the reaction mixture was stirred for 32 hr at 20 °C.  LC / MS (ES8260-5-P1H) showed the ***compound 4*** was consumed completely, and mainly ***Dw-3-15*** (R.T. = 0.918 min, [M+Na] = 717.3). Then the reaction mixture was concentrated under reduced pressure to remove solvent. The residue was purified by prep-HPLC (column: Phenomenex Synergi Max-RP 250 * 50mm *10 um; mobile phase: [Water-ACN]; B%: 35%-75%, 24 min) to give 10.3 g white solid.  LC / MS (ES8620-5-P1L1) and ^1^H-NMR (ES8620-5-P1N1) showed the ***Dw-3-15*** (10.3 g, 14.61 mmol, 47.98% yield, 98.55% purity) was obtained as a white solid, and HPLC (ES8620-5-P1H5) showed the purity of 98.55%.

^1^H-NMR (400MHz, CHLOROFORM-d) ppm δ = 7.19 (br d, J=8.3 Hz, 1 H), 7.07 - 6.89 (m, 2 H), 5.85 (br d, J=9.8 Hz, 1 H), 5.48 (br d, J=5.5 Hz, 1 H), 5.13 (br d, J=13.3 Hz, 1 H), 4.90 - 4.74 (m, 2 H), 4.47 (br d, J=17.3 Hz, 1 H), 4.07 (br d, J=17.3 Hz, 1 H), 3.02 - 2.75 (m, 8 H), 2.65 - 2.53 (m, 1 H), 2.51 - 2.33 (m, 2 H), 2.11 - 1.99 (m, 2 H), 1.95 - 1.69 (m, 8 H), 1.68 - 1.61 (m, 1 H), 1.58 - 1.46 (m, 3 H), 1.44 (d, J=2.5 Hz, 4 H), 1.41 - 1.22 (m, 6 H), 1.09 - 0.94 (m, 4 H), 0.87 (d, J=7.0 Hz, 3 H)

LCMS: ES8620-5-P1H LCMS: ES8620-5-P1L1

HPLC: ES8620-5-P1H5 ^1^H NMR: ES8620-5-P1N1

**THE END**
